# Linking persistent negative symptoms to amygdala–hippocampus structure in first-episode psychosis

**DOI:** 10.1038/tp.2017.168

**Published:** 2017-08-08

**Authors:** C Makowski, M Bodnar, J J Shenker, A K Malla, R Joober, M M Chakravarty, M Lepage

**Affiliations:** 1 Douglas Mental Health University Institute, Verdun, QC, Canada; 2 McGill Centre for Integrative Neuroscience, McConnell Brain Imaging Centre, Montreal Neurological Institute, Montreal, QC, Canada; 3 Department of Psychology, McGill University, Montreal, QC, Canada; 4 Prevention and Early Intervention Program for Psychosis, Douglas Mental Health University Institute, McGill University, Montreal, QC, Canada; 5 Department of Psychiatry, McGill University, Montreal, QC, Canada; 6 Department of Biological and Biomedical Engineering, McGill University, Montreal, QC, Canada

## Abstract

Early persistent negative symptoms (PNS) following a first episode of psychosis (FEP) are linked to poor functional outcome. Reports of reduced amygdalar and hippocampal volumes in early psychosis have not accounted for heterogeneity of symptoms. Age is also seldom considered in this population, a factor that has the potential to uncover symptom-specific maturational biomarkers pertaining to volume and shape changes within the hippocampus and amygdala. T1-weighted volumes were acquired for early (*N*=21), secondary (*N*=30), non-(*N*=44) PNS patients with a FEP, and controls (*N*=44). Amygdalar–hippocampal volumes and surface area (SA) metrics were extracted with the Multiple Automatically Generated Templates (MAGeT)-Brain algorithm. Linear mixed models were applied to test for a main effect of group and age × group interactions. Early PNS patients had significantly reduced left amygdalar and right hippocampal volumes, as well as similarly lateralized negative age × group interactions compared to secondary PNS patients (*P*<0.017, corrected). Morphometry revealed decreased SA in early PNS compared with other patient groups in left central amygdala, and in a posterior region when compared with controls. Early and secondary PNS patients had significantly decreased SA as a function of age compared with patients without such symptoms within the right hippocampal tail (*P*<0.05, corrected). Significant amygdalar–hippocampal changes with age are linked to PNS after a FEP, with converging results from volumetric and morphometric analyses. Differential age trajectories suggest an aberrant maturational process within FEP patients presenting with PNS, which could represent dynamic endophenotypes setting these patients apart from their non-symptomatic peers. Studies are encouraged to parse apart such symptom constructs when examining neuroanatomical changes emerging after a FEP.

## Introduction

Negative symptoms are a cluster of symptoms that represent a disabling feature for many psychiatric and neurological conditions, characterized by the absence of goal-directed behaviour, and related cognitive and emotional states underlying motivation.^1, 2^ It is clear that for individuals who have experienced a first episode of psychosis (FEP), the manifestation of persistent negative symptoms (PNS) underlines a significant subgroup of patients with unmet therapeutic needs.^3, 4^ Early persistent negative symptoms (ePNS) are defined by the presence of anhedonia-asociality, alogia, affective flattening and/or avolition-apathy for at least six consecutive months in the absence of therapeutically significant levels of positive, extrapyramidal and/or depressive symptomatology. Patients presenting concurrently with the latter symptom cluster comprise patients with PNS due to *secondary* factors (sPNS), and are argued to be distinct from ePNS.^5^ Evidence suggests that there are pronounced cortical changes specific to ePNS compared with other FEP patients.^3, 6, 7^ Within limbic circuitry, the structure of the amygdala and hippocampus may be particularly informative, given the significant level of communication between these two structures^8, 9^ and external brain networks,^10, 11^ and their crucial roles in various cognitive processes^8, 12^ thought to be compromised in PNS patients.^5, 13^

The link between PNS and amygdalar–hippocampal (AG–HC) structure in FEP is yet to be examined longitudinally, although earlier work has examined relevant themes cross-sectionally.^6, 14^ There is a considerable amount of literature on volumetric differences in these structures, with evidence supporting lower hippocampal volumes in chronic schizophrenia,^15, 16^ albeit the strength and direction of this result is contested in earlier stages of the illness.^17^

The heterogeneity among psychosis samples may contribute to such inconsistencies. Studies have addressed this by subdividing patients into subgroups according to various characteristics. For instance, previous work has pinpointed lower left hippocampal volumes localised to the tail region in first-episode schizophrenia patients who do not achieve remission after 6 months of treatment.^18^ In relation to negative symptoms, several studies^19, 20^ have found a relationship between functional activation patterns in the amygdala and severity of affective flattening in schizophrenia. Symptom severity has also been shown to be associated with the size and shape of the amygdala in psychosis and mood disorders.^21, 22^ In particular, negative symptoms may be linked to hippocampal structure, as demonstrated recently in an investigation pinpointing CA1 atrophy to worsening negative symptoms.^23^ If these relationships hold true, one might expect that patients with ePNS may exhibit differential AG–HC structure in relation to their non-PNS peers.

In 2014, Arnett *et al.*^24^ discussed a later sociodevelopmental maturational period (‘emerging adulthood’), encompassing the age range of 18–29 years. This has vast implications for psychosis, given the emergence of a FEP within this time frame. It is feasible that neurobiological trajectories may be altered in PNS patients showing poor functional outcome, and alterations may emerge as a function of age. There has been a great deal of literature vetting for the neurodevelopmental hypothesis of schizophrenia, but surprisingly few studies emphasising age in these patient populations. One developmental study of emotional processing suggested longitudinal patterns of amygdalar–prefrontal (AG–PFC) connectivity with age (particularly implicating connections to anterior cingulate and medial prefrontal cortices), where during childhood (that is, ages 7–12), positive associations between AG–PFC connectivity strength and response to emotional faces were evident, followed by a progressive shift to negative associations in adulthood (that is, ages 19–25).^25^ This was postulated to reflect top–down inhibitory control of prefrontal regions on amygdalar function in response to emotional stimuli in normal development. The idea of aberrancies in these connections in schizophrenia being more closely related to negative symptoms is still speculative; however, previous work from our group has shown altered cortical trajectories in various prefrontal regions in ePNS with age,^7^ which provides a foundation to justify further investigation of highly interconnected limbic structure. At the level of the hippocampus, a recent review proposed that the underconnectivity of AG–PFC in schizophrenia underlying emotional/sociocognitive processing deficits may be linked to hyperactivation of the hippocampus.^26^ The hippocampus has been dubbed as a key anatomical region in the initiation of schizophrenia, with aberrations strongly supported by altered neurodevelopmental mechanisms.^27^ Thus, changes in structure of the amygdala and hippocampus in the early phase of psychotic disorders may provide important neurodevelopmental information differentiating subgroups of patients with different clinical profiles. Of note, differences with age might be best captured by methods aside from conventional volumetry, as demonstrated by Voineskos *et al.*^28^ Neurodevelopmental changes within subcortical structures have also been depicted by several other studies, including an investigation of a child-onset schizophrenia sample.^29, 30^

The current study combines the power of a longitudinal design conducted at a single site with clinically well-characterised patients, with minimal to no prior exposure to antipsychotic medication, to begin to address pertinent questions of differential limbic structure trajectories in subgroups of FEP. To test for specificity of results to ePNS, we also compare against a subgroup of non-PNS patients with sPNS. It is hypothesised that ePNS patients will have lower volumes within the amygdala and hippocampus compared with both sPNS and non-PNS patients and controls. Merging knowledge from the AG–HC circuitry^31, 32^ and previously reported results,^18, 33^ morphometric differences within the amygdala are postulated to be localised at both lateral and medial aspects of the amygdala, involved in sensory integration and control of outputs, respectively. For the hippocampus, differences are hypothesised to be associated with the output region of the structure (for example, subiculum; closer to the hippocampal tail), as this region has previously been pinpointed in first-episode psychosis. Finally, we expect morphometric differences to vary as a function of age between ePNS and other FEP subgroups.

## Materials and methods

### Participants

Ninety-five patients and forty-four controls were included. See Supplementary Figure 1 for visualisation of the sample distribution by age. All patients were recruited from the Prevention and Early Intervention Program for Psychoses (PEPP-Montréal), at the Douglas Institute, and were part of a longitudinal naturalistic outcome study. PEPP is a specialised early intervention service for individuals between the ages of 14 and 35 who are experiencing a FEP within a local catchment area of Southwest Montréal, Canada. Details are outlined elsewhere.^34^ The programme involves a comprehensive approach with intensive medical and psychosocial interventions provided within the context of a modified assertive case management model. Given the nature of the study design, no statistical methods were used to predetermine sample sizes.

### Neuroimaging component

The neuroimaging study began in 2003, in which patients partook in three scheduled visits: baseline, 1-year follow-up (FUP1) and 2-year follow-up (FUP2). Inclusion criteria included the following: age above 18 years, diagnosis of affective or non-affective psychosis, IQ>70, no past antipsychotic medication treatment for more than 1 month before entry to PEPP, no major medical disorders and sufficient stability for the scanning procedure. Note that, although exposure to antipsychotic medication was restricted before acceptance to PEPP, most patients were prescribed antipsychotic medication before their first scan, and thus some patients did in fact have more than 1 month of cumulative exposure to antipsychotic medication for the neuroimaging portion of the study. Exclusion criteria include the following: a history of neurological illnesses and head trauma resulting in loss of consciousness that could affect cognition, presence of neurological disorder as by medical record examination, lifetime diagnosis of substance dependence and/or any potential contraindication for the magnetic resonance imaging scan. See Supplementary Methods for detailed information on patients excluded from the neuroimaging study.

Non-clinical controls were recruited through advertisements within the same local catchment area. In addition to exclusion criteria listed for FEP patients, controls were excluded if they had any current/past history of Axis I disorders, and/or a first-degree relative suffering from a schizophrenia spectrum disorder. All participants provided written informed consent, and the protocol was approved by the Research Ethics Board of the Douglas Mental Health University Institute and the McGill University Faculty of Medicine.

### Clinical assessment and demographic data

Diagnosis was made using the Structured Clinical Interview for the Diagnostic Statistical Manual, Version IV (SCID-IV),^35^ performed by a trained interviewer and confirmed by a research clinician psychiatrist. Depression was assessed with the Calgary Depression Scale for Schizophrenia.^36^ Positive and negative symptoms were assessed with the Scale for the Assessment of Positive Symptoms (SAPS)^37^ and Scale for the Assessment of Negative Symptoms (SANS).^38^ Antipsychotic medication dosages were converted to chlorpromazine equivalents according to the literature,^39^ and multiplied by percent medication adherence.^40^ Parental socioeconomic status,^41^ handedness^42^ and full-scale IQ^43, 44^ were assessed for both controls and patients.

Following our previous work,^5, 7^ early PNS were defined according to the following criteria: (1) global rating of moderate or more on at least one negative symptom as measured by the SANS; (2) global rating of mild or less on all positive symptoms as measured by the SAPS; (3) a total score of four or less on the Calgary Depression Scale for Schizophrenia (CDSS); (4) absence of extrapyramidal symptoms requiring anticholinergic treatment; and (5) all above criteria are maintained for a period of at least 6 months.^4, 5^ Patients were classified as having PNS due to secondary factors if criteria 2, 3 and/or 4 were not met. Specifically, if moderate or worse levels of delusions or hallucinations were present from month 3 to 6 or from month 6 to month 9, severe levels of negative symptoms were considered secondary.

### Magnetic resonance imaging acquisition

All scanning procedures were carried out at the Montreal Neurological Institute on a 1.5-T Siemens Sonata scanner. T1-weighted volumes were acquired using a three-dimensional gradient echo pulse sequence with sagittal volume excitation (TR)=22 ms, echo time (TE)=9.2 ms, flip angle=30, 180 1-mm contiguous sagittal slices). The rectangular field of view for the images was 256 mm (superior-inferior (SI)) and 204 mm (anterior-posterior (AP)).

### Post-processing: MAGeT-brain

Amygdalar and hippocampal structures were extracted bilaterally using the Multiple Automatically Generated Templates (MAGeT)-Brain algorithm^28, 30, 45^ (https://github.com/CobraLab/MAGeTbrain). This technique utilises a limited number of high-resolution atlases that have been manually segmented as described previously (amygdala;^46^ hippocampus;^47^ https://github.com/CobraLab/atlases). Extensive validation of MAGeT has been done previously, as shown in several references from our group,^45, 48^ which have also included subsets of the described patient sample here, with data acquired on a 1.5-T scanner. Segmentations were also submitted to the shape morphometric branch of MAGeT-Brain, yielding local vertex-wise surface area (SA) maps for each subject. Information about MAGeT-Brain processing and quality control is detailed in Supplementary Methods. An example for a representative candidate of segmentation of the amygdala and hippocampus, and of vertex wise SA are depicted in Supplementary Figures 2 and 3, respectively.

### Statistical analyses

Demographic and clinical variables (with a single time point) were analysed with one-way analyses of variance for continuous variables or *χ*^2^-ratio tests for nominal variables. For IQ, an analysis of covariance was used to covary for test version. SAPS/SANS sums of item scores between FEP subgroups were assessed across clinical time points with Generalised Estimating Equations. Antipsychotic dosages, CDSS scores and the time period in months between scan and nearest symptom evaluation were assessed between the three patient groups at each scan time point, using one-way analyses of variance for normally distributed variables, and Kruskall–Wallis*H*-tests for non-normally distributed variables. Analyses of clinical variables were conducted using PASW Statistics 21 (SPSS, 2009, Chicago, IL, USA) and were two-tailed with a critical *P*-value of 0.05.

### Neuroanatomical analyses: volume

For scans that passed quality control (see Supplementary Methods), volumetric differences between FEP subgroups and controls were assessed using linear mixed effects models applied to each structure and hemisphere separately using Matlab (2015a). Gross volumetric differences in structure were assessed with the following model:


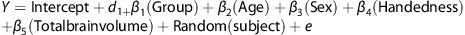


where *Y* represents whole left/right amygdalar/hippocampal volume, *d*_1_is the random within-subjects effect, *β*_1–5_ represents regression coefficients and *ε* is residual error. Linear age effects were then examined separately by adding the following term to the above model: ‘*β*_6_(group × age)’. To control for multiple comparisons, the false discovery rate (FDR) procedure was used with *q*=0.05, which limits the expected proportion of incorrectly rejected null hypotheses to 5%.^49^

### Neuroanatomical analyses: SA

To assess differences in shape morphometry between groups, statistics were performed across all vertices of bilateral amygdalar and hippocampal surfaces using the SurfStat toolbox within Matlab (http://www.math.mcgill.ca/keith/surfstat/). Each hemisphere was assessed separately, using an equivalent mixed effects model as described in the previous section, covarying for total SA of the structure by hemisphere in place of total brain volume (*β*_5_ (Total SA)). Similarly, the main effect of group was first tested, followed by linear age × group interactions. For all analyses, statistical maps were thresholded and multiple comparisons were taken into account using random field theory for non-isotropic images,^50^ limiting the chance of reporting a false positive finding to below *P*=0.05.

### Supplementary linear mixed effects models with altered covariates

Four additional models were tested with altered covariates, to explore effects of different variables, in addition to the chosen covariates of sex, handedness and total brain volume/total SA. These four altered models were as follows: (A) covarying for diagnosis, (B) covarying for antipsychotic medication in the FEP patient sample only; as described above, antipsychotic dosages were converted to chlorpromazine equivalents and took into account medication adherence, (C) removal of sex and handedness and (D) covarying for IQ (note two controls were excluded from this analysis, given missing IQ information). The rationale behind analyses C was motivated by the fact that our groups did not significantly differ on sex and handedness. These variables were kept in the main model presented in this manuscript, given the well-documented and clear impact of sex and handedness on neuroanatomy.^51, 52, 53^ However, recent evidence has not found support for the effects of handedness on cerebral anatomy.^54^ With respect to sex differences, Pruessner *et al.*^55^ reported no sex differences in amygdalar and hippocampal volumes. Thus, it is of interest to investigate how significant findings may be altered when these variables are removed from the model.

## Results

### Sociodemographic and clinical

In the FEP group, baseline scans were performed on average 4.1 (s.d.=1.9) months after entry to PEPP. For the entire group, including controls, interscan intervals were ~13.1 (s.d.=1.3) months between baseline and FUP1, and 12.5 (s.d.=1.7) months between FUP1 and FUP2. Nine participants (six FEP and controls) were not scanned at FUP1 but were scanned at FUP2; average interscan interval was 26.7 (s.d.=3.1) months between baseline and FUP2.

The groups did not significantly differ in sex ratio, handedness, parental socioeconomic status or age at scanning time (see Table 1). However, controls significantly differed from all patient groups on Full-Scale IQ and years of education. Within the three patient groups, there were no significant differences in CDSS scores or time elapsed between the magnetic resonance imaging scan and symptom evaluation. As expected, the sPNS patient subgroup had significantly higher SAPS totals compared with the ePNS and non-PNS subgroups across baseline and 1-year follow-up. In addition, the ePNS and sPNS subgroups had significantly higher SANS totals compared with the non-PNS subgroup across all time points. See Supplementary Table 1 and Supplementary Figure 4 for breakdown of SAPS/SANS scores across clinical time points and relevant statistics. FEP subgroups differed in distribution of diagnosis, with a higher proportion of non-PNS diagnosed with affective psychotic disorders (major depression, bipolar), and higher proportions of schizophrenia/schizophreniform diagnoses in the sPNS and ePNS subgroups. In addition, amount of antipsychotic prescribed at the second scanning time point was significantly higher for sPNS patients compared with non-PNS patients; thus, diagnosis and antipsychotic medication were included as covariates in supplemental analyses (see Supplementary Figure 5).

### Amygdalar and hippocampal volumetry

**Figure 1:**
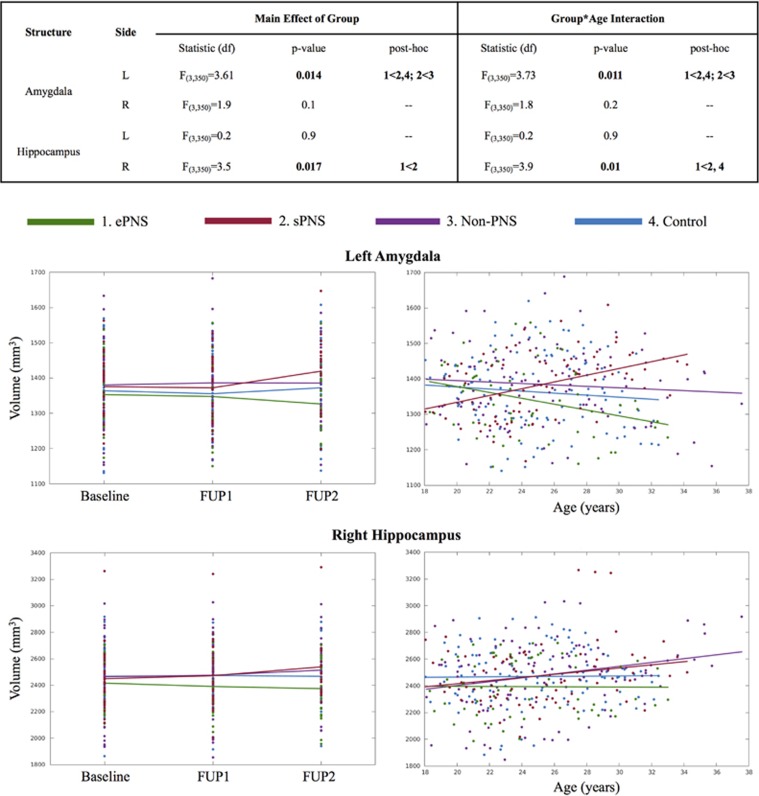
Amygdalar and hippocampal volumes: significant group main effects and group × age interactions. Table presents statistics from linear mixed effects analyses. Significant results that survived FDR correction for multiple comparisons are indicated in bold. *Post hoc* contrasts were based on the four groups: (1) ePNS, (2) sPNS, (3) non-PNS and (4) Controls. Significant group and group × age contrasts are depicted in the corresponding graphs below the table. ePNS, early persistent negative symptoms; FUP, follow-up; sPNS, persistent negative symptoms due to secondary factors.

Descriptive statistics of left/right amygdalar and hippocampal volumes adjusted for age, sex, total brain volume and handedness are outlined in Table 2. After FDR correction, linear mixed models revealed significant group differences for right amygdalar volumes (F_3,350_=3.61, *P*=0.014) and hippocampal volumes (F_3,350_=3.5, *P*=0.017). Significant effects also emerged for age × group interactions for left amygdalar volumes (F_3,350_=3.73, *P*=0.011), as well as the right hippocampus (F_3,350_=3.9, *P*=0.010). *Post hoc* tests (all *P*<0.05) showed that within the left amygdala, the sPNS group had a significantly different and positive correlation with age compared with ePNS patients and controls. The non-PNS group also had a significantly different slope with age compared with ePNS patients. For the right hippocampus, the ePNS group had a significantly different negative correlation with age compared with sPNS patients and Controls. No significant group or age × group differences emerged for the right amygdala or left hippocampus (Figure 1).

### Hippocampal and amygdalar shape morphometry—vertex-wise results

**Figure 2:**
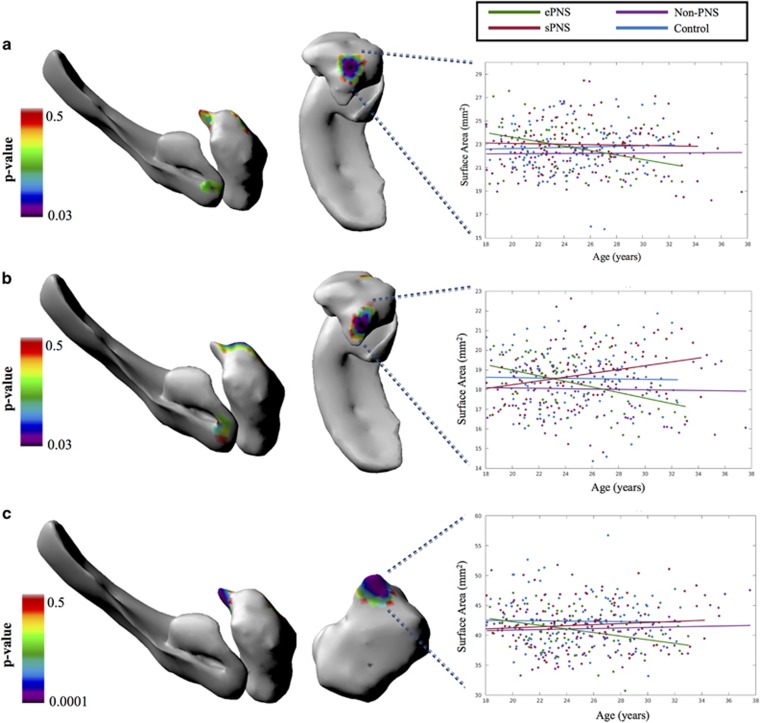
Significant group × age interactions in surface area of the left hemisphere. Statistical maps overlaid on left hippocampal and amygdalar 3D surface renderings represent RFT-corrected *P*-values. Surface area metrics from clusters with a corrected *P*-value less than 0.05 were extracted and plotted against age for each group. (**a**) Significantly decreased surface area with age in the ePNS group compared with non-PNS within a central/anterior region of the left amygdala (292 df, *P*=0.03). Comparison of regression slopes with age reveals similar effects comparing with controls as well (F_(3,350)_=5.01, *P*=0.002; *post hoc* ePNS<Controls *P*=0.0011). (**b**) Significantly decreased surface area with age in the ePNS group compared with sPNS in a more dorsal region (compared with A) of the central amygdala (292 df, *P*=0.03). Further comparison with other groups revealed significant differences in regression slopes with age (F_(3,350)_=4.43, *P*=0.0045), such that the ePNS had a significantly reduced relationship between age and surface area in this cluster compared with both non-PNS and Controls (*P*⩽0.01). (**c**) Significantly decreased surface area with age in the ePNS group compared with Controls in a posterior (centromedial) portion of the amygdala (298 df, *P*=0.0001). Mixed effects statistics for comparison of regression slopes yielded F_(3,350)_=4.19, *P*=0.006, where no other contrasts apart from Controls versus ePNS were significant. No significant interaction effects with age emerged for the left hippocampus. Orientation: from left to right, surfaces depict left medial view and dorsal view of the hippocampus and amygdala, with the exception of **c**, where the dorsal view has been replaced by a posterior view of the amygdala for better visualisation of the significant cluster. df, degree of freedom; ePNS, early persistent negative symptom; RFT, random field theory; sPNS, persistent negative symptoms due to secondary factors.

**Figure 3:**
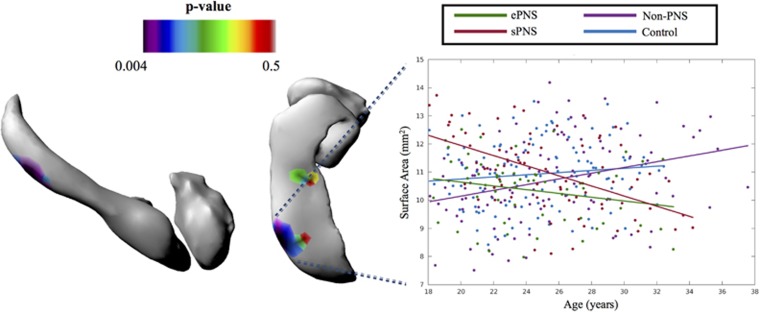
Significant group × age interactions in surface area of the right hemisphere. Statistical maps overlaid on left hippocampal and amygdalar 3D surface rendering represent RFT-corrected *P*-values. Significant cluster emerged with increased surface area with age in non-PNS compared to sPNS and ePNS (*P*<0.01) in a posterior/ventral portion of the hippocampus. Further comparison to other groups revealed significant differences in regression slopes with age (F_(3,350)_=7.04, *P*<0.001), such that the ePNS group had a significantly negative relationship between surface area in this cluster and age compared to sPNS and non-PNS (*P*⩽0.001). In addition to sPNS differing from non-PNS, sPNS patients also had a significantly different positive slope compared with Controls (*P*<0.001). No significant interaction effects with age emerged for the right amygdala. Orientation: from left to right, surfaces depict right lateral view, followed by ventral view of hippocampal and amygdalar structures. ePNS, early persistent negative symptoms; RFT, random field theory; sPNS, persistent negative symptoms due to secondary factors.

There were no significant main effects of group for either structure bilaterally. However, significant findings emerged with the age × group interaction. For the left amygdala, the following contrasts and regions had significantly different SA trajectories with age comparing groups on vertex-wise SA: (1) ePNS<non-PNS within a central/anterior cluster (Figure 2a), (2) ePNS<sPNS and Controls in a more dorsocentral region (Figure 2b) and (3) ePNS<Controls within a posterior/centromedial portion (Figure 2c). For the right hippocampus, a significant cluster emerged in a portion of the hippocampal tail comparing SA trajectories with age between non-PNS patients and the other FEP subgroups; specifically, the ePNS group had a significant negative relationship with age in this cluster compared with sPNS and non-PNS subgroups, and the sPNS group also exhibited a significantly different and opposite trajectory compared with non-PNS patients and controls (*P*⩽0.001; Figure 3). No significant age × group interactions were found for SA across the left hippocampus or right amygdala. Controlling for diagnosis, antipsychotic medication and IQ did not significantly change the interpretation of results. Similarly, removing handedness and sex as covariates in the linear mixed effects model did not alter results, with the exception of one SA cluster of the left central amygdala, which did not survive correction for multiple comparisons with random field theory after removing sex and handedness as covariates, namely when comparing age trajectories between ePNS and non-PNS patients. See Supplementary Table 2 and Supplementary Figure 5 for results with altered covariates for volumetry and shape morphometry, respectively.

### Hippocampal and amygdalar shape morphometry—*post hoc* region-of-interest results

For the four significant clusters that emerged through a vertex-wise investigation of SA described above, total SA values were extracted for each of the regions and regression slopes were calculated for each of the four groups (ePNS, sPNS, non-PNS and Controls), to see whether any other groups differed at the regional level. For the first central/anterior amygdalar cluster, in addition to differing from non-PNS patients, the ePNS group had significantly different regression slopes from Controls (Omnibus: F_(3,350)_=5.01, *P*=0.002; *post hoc* ePNS<Controls *P*=0.0011; Figure 2a). For the second amygdalar region (dorsal to the first), ePNS had a significantly reduced relationship between age and SA in this cluster compared with both non-PNS and Controls (Omnibus: F_3,350_=4.43, *P*=0.0045; *post hoc* *P*⩽0.01), in addition to the previously reported vertex-wise difference between ePNS and sPNS (Figure 2b). Finally, the last significant amygdalar cluster, localised more posteriorly and centromedially, did not uncover any additional significant relationships apart from the previously reported difference between ePNS and Controls (Omnibus: F_3,350_=4.19, *P*=0.006; Figure 2c).

For the single significant right hippocampal cluster, further*post hoc* analyses of regression slopes revealed additional differences between sPNS patients and controls (Omnibus: F_3,350_=7.04, *P*<0.001; *post hoc* sPNS<Control, *P*<0.001; Figure 3).

## Discussion

The current study provides evidence for changes in AG–HC structural trajectories, specifically in FEP patients presenting with PNS. Volumetric findings within the left amygdala and right hippocampus indicated that ePNS patients had significantly different/decreased relationships with age compared with non-PNS patients and controls, in addition to having significantly reduced volumes within these structures. SA findings were similarly lateralised, where the most prominent direction of findings emerged with significant contraction with age in ePNS across several amygdalar regions and a posterior hippocampal cluster. Furthermore, the sPNS group showed significantly decreased SA with age within the latter hippocampal region in opposition to the notable expansion with age examined in non-PNS patients and controls. Noteworthy, non-PNS patients never differed from healthy controls. Results remained largely unaltered when covarying for IQ, diagnosis and antipsychotic dosage, and removing sex and handedness from the model.

The differential and striking trajectories uncovered in relation to negative symptom presentation within AG–HC structure encourage further exploration of dynamic brain changes in different psychiatric samples, as others have suggested.^56, 57^ Nacewicz *et al.*^58^ explored such age effects on the amygdala in an autistic sample, and found significantly lower amygdalar volumes in older individuals with autism, not unlike the amygdalar trajectories found within our ePNS group. Given the parallels that can be drawn between flattened affect and social impairments in autism with symptom presentation in ePNS, the amygdala represents a plausible target for future transdiagnostic work. At the level of the hippocampus, associations have been previously drawn between lower hippocampal volumes and poor functioning in FEP,^59^ lending support to our findings of decreased right hippocampal volumes in patients with ePNS and corresponding negative trajectories with age.

Notably, only age × group interactions on specific regions of AG–HC shape morphometry-yielded significant results, concordant with previous claims that differences in SA morphology may represent a dynamic and neurodevelopmental endophenotype.^29, 30, 60, 61, 62, 63^ Few studies have looked at AG–HC shape morphology in psychosis, although relevant objectives were investigated in the work of Qiu *et al.*^22^ The authors found significant surface alterations within the left hippocampal tail and right hippocampal body, with first-episode schizophrenia patients exhibiting greater inward deformations compared with first-episode mania and controls. This is consonant with the significant inward deformations consistently seen in our ePNS group, containing a higher distribution of patients diagnosed with schizophrenia/schizophreniform (as opposed to an affective disorder). In fact, controlling for diagnosis in our analyses strengthened the statistical significance of shape deformation clusters.

The consistent lateralisation of results observed across volumetric and morphometric analyses deserves discussion. Although studies in FEP and schizophrenia have uncovered differences bilaterally, many findings in psychosis have been skewed to the left hemisphere.^18, 64, 65, 66^ In contrast, our findings within the hippocampus were right-lateralised and were specific to patients with PNS. Witthaus *et al.*^67^ reported similarly lateralised findings in an investigation of patients at ultra-high risk for psychosis and those who transitioned to having a FEP. Specifically, this study pinpointed lower volumes in a subset of ultra-high risk patients who transitioned to psychosis within the left amygdala, and lower right hippocampal volumes in FEP patients. These consistent findings at similar stages of psychosis suggest that lateralisation of limbic structural volume differences may reflect an early biomarker underlying the manifestation of psychosis and subsequent negative symptomatology. The localisation of deformation differences also overlapped with our initial hypotheses, where significant differences emerged in a medial region of the amygdala, a region posited to have ‘striatal-like’ features with GABAergic-containing neural circuitry.^32, 68^ We also observed hippocampal morphometric differences closer to the output region of the structure, in line with previous studies.^22, 31, 33^ Although not initially hypothesised, significant differences were uncovered within central regions of the amygdala, which has an integral role in forming associations between stimuli on the basis of their motivational salience, ultimately shaping emotional behaviours.^69, 70^ Thus, aberrancies in the development of these key regions involved in emotion and motivational behaviours may contribute to the differential expression of negative symptoms exhibited by ePNS and sPNS patients, although further work is needed to understand the mechanisms underlying the manifestation of symptoms in these two groups.

Another noteworthy point is the significantly different trajectories uncovered within the sPNS group, particularly within the hippocampus. We had initially hypothesised that the ePNS group would show the most significant changes, and we had not expected such marked effects in the sPNS group, meriting additional dialogue on the potential effect of positive symptoms on the limbic structure, and differential changes with age. Links between hippocampal shape and positive symptomatology were recently addressed by Mamah *et al.*,^33^ in which higher levels of disorganised positive symptoms were significantly correlated with surface contraction in the lateral CA1 hippocampal subregion. Depressive symptomatology also may have contributed to results within the sPNS group. For instance, previous work^71^ has suggested that amygdalar volume reductions seem to be specific to the intersection of psychosis and depression. Other work has corroborated evidence for hippocampal shape changes in depression.^72^ Further longitudinal investigation of positive and depressive symptomatology in relation to amygdalar and hippocampal structure is warranted to disentangle the specific contributions of different symptom domains.

Several studies have refuted the idea of progressive structural changes in the hippocampus and amygdala after the onset of psychosis.^17, 73, 74^ However, our findings suggest that null findings may be a result of pooling together patients into a unitary group and simply comparing with healthy controls, which would be similarly found in our sample if our three FEP subgroups were merged. The age window at which a FEP is experienced has large consequences for the social developmental stage of the individual, and it comes as no surprise that these effects may be manifested differentially with age at the level of limbic structure. Neurodevelopmental models of psychosis-related disorders are increasingly beginning to interlace psychosocial and biological factors into a coherent model to facilitate treatment,^75^ which requires further understanding of potential gene × environment interactions on neurobiology to help us fully understand the psychological and biological mechanisms contributing to PNS following a FEP.

It is worth discussing the chosen categorical approach, as opposed to the often-used ‘dimensional’ approach of regressing symptom severity against neuroanatomical measures. Although meaningful information can certainly be derived by the latter approach, symptom data are often not normally distributed, and the amount of clinical information used in such brain–behaviour relationships is often limited by the imaging data. Given that the current study design had a greater number of clinical time points (that is, 5+) compared with imaging time points (that is, 2–3), regression analyses would have restricted the presented analysis to the available imaging data, and important dynamic information regarding the longitudinal course of symptoms across different domains would have been lost. Thus, the adopted approach capitalises on the clinical data available in linking symptoms to neuroanatomical trajectories within the amygdala and hippocampus. Finally, our findings suggest that the resultant subgroups of patients do indeed seem to have distinct biological underpinnings in AG–HC structure, and such an approach in defining patient subgroups independent of diagnostic categories may inform and/or contribute to pending changes in diagnostic categories that have often been criticised for lacking biological validity. Although future work is certainly required to gain more confidence in the validity of the proposed subgroups, this approach holds promise in bringing to the forefront meaningful clinical subtypes of patients who have experienced a FEP and addressing the clinical picture surrounding negative symptom presentation.

The current study offers several strengths and limitations. Recruitment strategies have been optimised for this large sample of FEP patients such that all patients were recruited from a single well-defined catchment area in the absence of other competing services. Furthermore, a wealth of longitudinal data is available for these patients, including data from structured clinical assessments to complement neuroimaging data. However, there are inherent limitations to the manner by which early and secondary PNS groups were separated. Given that the latter group exhibits treatment resistance, and arguably, poor outcome similarly to ePNS patients, future investigations should try to incorporate additional behavioural and clinical data to disentangle neurobiological findings. It is possible that these two subgroups of patients have overlapping neuroanatomical features that were not directly addressed by this study. There was also an uneven drop-out rate among the FEP subgroups for the neuroimaging portion of the study, with the highest attrition observed in the sPNS group. Finally, imaging was acquired on a 1.5-T scanner, which prevented us from reliably resolving hippocampal subfield structure, an emerging interest in the study of neuropsychiatric disorders.^65, 76, 77, 78, 79^

There have been a wide array of findings pinpointing aberrant AG–HC structure in psychotic disorders, with scant research looking at specific symptom constructs in psychosis, and further localising changes with surface morphometry. The current study addresses these gaps in the literature and elucidates differential volumetric and shape morphometric trajectories with age within lateralised regions of the amygdala and hippocampus, in relation to persistent negative symptoms in psychosis. These findings suggest potential neurodevelopmental aberrations that coincide with negative symptom presentation, and could represent dynamic endophenotypes underlying patient subgroups within heterogeneous first-episode psychosis populations. As alluded to, current pharmacological interventions have poor efficacy on negative symptoms, and a better understanding of the biological mechanisms and anatomical/functional consequences underlying such symptom presentation may allow for better and more targeted design of future medications. In parallel with an improved description of what is occurring at the neural level, it will be important to test concrete behavioural measures, such as verbal memory, to unravel the effects of therapeutic interventions in early psychosis and other implicated neuropsychiatric conditions on potential improvement of negative symptoms. Improved models of brain–behaviour relationships alongside clinical descriptors of negative symptoms hold promise in translational research and dissipating the status of negative symptoms as a largely unmet therapeutic need in psychotic disorders, especially for more prevalent domains of negative symptoms encompassing avolition, asociality and anhedonia.

## Figures and Tables

**Table 1:** Demographic and clinical information for longitudinal sample

		*FEP*					
			*Non-ePNS*				
		*ePNS*	*sPNS*	*Non-PNS*	*Controls*	*Statistic*_*(df)*_	P*-value*
*General demographics*	*N*(+ subset with three scans)	21 (18)	30 (15)	44 (27)	44 (24)		
	Male, *N* (%)	15 (71.4)	21 (70.0)	31 (70.5)	25 (56.8)	*χ*^2^_(3)_= 2.5	0.5
	Education in years	11.1 (2.5)	11.6 (2.4)	12.7 (2.4)	14.2 (2.5)	F_(3,138)_=10.4	<0.001^a^
	Socioeconomic status	3.4 (1.0) [16]	3.4 (1.1) [29]	3.0 (1.0) [42]	3.4 (0.9) [41]	*χ*^2^_(3)_= 6.1	0.1
	Right handed, *N* (%)	17 (81.0)	25 (83.3)	38 (86.4)	38 (86.4)	*χ*^2^_(3)_= 0.5	0.9
	Full scale IQ^b^	96.9 (15.3)	97.8 (15.3)	100.3 (15.3)	111.5 (15.3) [42]	F_(3,136)_=7.1	<0.0001^a^
	Diagnosis**, *N* (%)					*χ*^2^_(6)_= 13.1	0.017
	Schizophrenia/schizophreniform^c^	16 (76.2)	26 (86.7)	24 (54.5)			
	Affective disorder	3 (14.3)	1 (3.3)	15 (34.1)			
	Delusional disorder	0 (0)	1 (3.3)	2 (4.5)			
	Psychosis not otherwise specified	2 (9.5)	2 (6.7)	3 (6.8)			
							
*Scan 1*	Age	23.2 (3.6)	24.5 (4.0)	4.6 (0.7)	23.8 (3.5)	F_(3,138)_=1.0	0.4
	Window |Scan−Symptom Eval| (months)	0.6 (0.4)	0.8 (0.5)	0.7 (0.6)		F_(2,94)_=0.5	0.6
	SAPS global	3.6 (3.8)	6.6 (3.9)	2.4 (2.6)		*χ*^2^_(2)_= 22.2	<0.0001^d^
	SANS global	9.6 (2.9)	9.0 (3.5)	5.9 (3.2)		F_(2,94)_=13.3	<0.0001^e^
	CDSS	2.4 (2.7)	3.1 (3.2)	1.7 (2.5) [43]		*χ*^2^_(2)_= 3.4	0.2
	CPZ equivalent (in mg)	758.4 (671.3)	965.9 (844.6)	774.2 (707.9)		*χ*^2^_(2)_=9.7	0.6
	Adherence (%)	86.6 (21.3)	87.9 (19.1)	84.6 (27.5)		*χ*^2^_(2)_= 0.2	0.9
	Antidepressant, *N*(%)	2 (9.5)	5 (16.7)	9 (20.5)			
	Benzodiazepine, *N* (%)	1 (4.8)	4 (13.3)	2 (4.5)			
	Anticholinergic, *N* (%)	1 (4.8)	5 (16.7)	6 (13.6)			
	Mood stabiliser, *N* (%)	1 (4.8)	0 (0)	7 (15.9)			
							
*Scan 2*	*N*	19	28	41	41		
	Age	24.3 (3.8)	25.5 (4.1)	25.6 (4.3)	24.7 (3.4)	F_(3,128)_=0.7	0.5
	Window |Scan−Symptom Eval| (months)	1.8 (1.5)	2.1 (1.7)	1.8 (1.2)		*χ*^2^_(2)_= 0.3	0.9
	SAPS global	2.7 (2.5)	5.7 (4.0)	1.6 (2.5)		*χ*^2^_(2)_= 24.3	<0.0001^d^
	SANS global	8.4 (3.5)	7.9 (3.6)	3.3 (3.2)		F_(2,87)_=22.8	<0.0001^e^
	CDSS	1.0 (1.5)	1.9 (3.0) [27]	1.4 (2.7)		*χ*^2^_(2)_= 1.7	0.4
	CPZ equivalent (in mg)	2875.2 (2059.7)	4434.7 (3337.6)	2656.8 (2187.0)		*χ*^2^_(2)_=10.3	0.006^f^
	Adherence (%)	87.0 (16.0)	80.0 (19.9)	81.1 (25.5)		*χ*^2^_(2)_=1.3	0.5
	Antidepressant, *N* (%)	4 (23.5) [17]	6 (22.2) [27]	10 (25.0) [40]			
	Benzodiazepine, *N*(%)	0 (0) [16]	3 (11.1) [27]	1 (2.4)			
	Anticholinergic, *N* (%)	1 (6.3) [16]	1 (3.7) [27]	2 (4.9)			
	Mood stabiliser, *N*(%)	2 (12.5) [16]	0 (0) [27]	5 (12.2)			
							
*Scan 3*	*N*	20	17	29	27		
	Age	25.5 (3.7)	26.2 (3.7)	26.3 (4.4)	26.9 (3.5)	F_(3,92)_=0.5	0.6
	Window |Scan−Symptom Eval| (months)	1.0 (1.9)	0.4 (0.5)	0.5 (0.7)		*χ*^2^_(2)_= 0.8	0.7
	SAPS global	3.0 (3.0)	4.1 (4.1)	2.0 (2.4)		*χ*^2^_(2)_= 3.1	0.2
	SANS global	7.2 (3.6)	5.6 (4.0)	3.0 (3.5)		*χ*^2^_(2)_= 16.9	<0.0001^e^
	CDSS	2.5 (3.3) [18]	2.1 (2.5)	1.6 (2.1) [28]		*χ*^2^_(2)_= 0.7	0.7
	CPZ equivalent (in mg)	4216.5 (3906.2)	6753.8 (6368.0)	5177.0 (4994.3)		*χ*^2^_(2)_= 2.0	0.4
	Adherence (%)	78.4 (26.2)	78.3 (27.9)	77.1 (28.7)		*χ*^2^_(2)_= 0.05	0.98
	Antidepressant, *N* (%)	5 (26.3) [19]	3 (21.4) [14]	3 (11.5) [26]			
	Benzodiazepine, *N*(%)	0 (0) [19]	0 (0) [14]	0 (0) [26]			
	Anticholinergic, *N* (%)	2 (10.5) [19]	0 (0) [15]	0 (0) [27]			
	Mood stabiliser, *N* (%)	1 (5.3) [19]	1 (7.1) [14]	6 (25.0) [24]			

Abbreviations: CDSS, Calgary Depression Scale for Schizophrenia; CPZ, chlorpromazine; ePNS, early persistent negative symptom; FEP, first-episode of psychosis; FUP, follow-up; SAPS/SANS, Scale for Assessment of Positive/Negative Symptoms; sPNS, persistent negative symptoms due to secondary factors.

General demographics for whole sample are presented, followed by information corresponding to each scan. All data represented as mean (s.d.), unless otherwise specified. Levene’s test revealed no significant differences in variance between subgroups. Square brackets [] include adjusted sample size included in statistical analysis because of missing data points. All antipsychotic totals are presented as cumulative chlorpromazine equivalents in mg, as prescribed by a psychiatrist, and are reported along with corresponding medication adherence percentages. SAPS/SANS totals are presented as the mean scores of the sum of item-level scores. Note that ‘SANS total’ excludes the ‘attention’ subscale.

a *Post hoc* comparisons showed that controls differed from all FEP patient groups in years of education (*P*<0.005) and IQ (*P*<0.01). IQ differences were covaried by test version. No differences existed between patient groups.

b IQ means and s.d. are presented as adjusted values, covaried by test version (WAIS-III versus WASI). There was no difference between different test versions on IQ (F_1,136_=0.9, *P*=0.3).

c Assessed using Fisher’s exact test of independence.

d Tukey’s *post hoc* comparisons revealed significant differences in SAPS scores between sPNS and other two patient groups (*P*<0.005).

e Tukey’s *post hoc* comparisons revealed significant differences in SANS scores between ePNS and sPNS and remaining non-PNS patients (*P*<0.001) for Scans 1 and 2. For Scan 3, non-PNS still significantly differed from ePNS (*P*=0.001), but there was only a trend-like difference between non-PNS and sPNS (*P*=0.08).

f *Post hoc* analyses indicated that sPNS patients were prescribed significantly more antipsychotic medication (in CPZ equivalent dosage) cumulatively compared with non-PNS patients at Scan 2 (*P*=0.02), and was still significant when taking into consideration medication adherence (multiplying CPZ equivalent by percent adherence), with *χ*^2^_(2)_=6.2, *P*=0.046 (*post hoc* sPNS>non-PNS *P*=0.03). No significant differences emerged between the ePNS group and other FEP subgroups at Scan 2.

**Table 2:** Amygdalar and hippocampal volumes: descriptives

			*FEP*			
				*Non-ePNS*		
*Time point*	*Structure*	*Side*	*(1) ePNS*	*(2) sPNS*	*(3) Non-PNS*	*(4) Controls*
Baseline (scan 1)	Amygdala	L	1386.9 (22.6)	1358.7 (18.6)	1362.2 (15.4)	1377.4 (15.5)
		R	1404.5 (22.0)	1370.5 (18.1)	1381.7 (15.0)	1384.6 (15.0)
	Hippocampus	L	2488.3 (52.0)	2452.1 (42.8)	2517.4 (35.4)	2536.1 (35.5)
		R	2471.0 (52.7)	2421.4 (43.4)	2433.9 (35.9)	2490.9 (36.0)
						
FUP1 (scan 2)	Amygdala	L	1384.6 (23.4)	1359.9 (19.0)	1365.7 (15.8)	1367.3 (15.8)
		R	1398.5 (22.9)	1362.9 (18.6)	1383.7 (15.4)	1395.8 (15.5)
	Hippocampus	L	2486.5 (52.2)	2485.6 (42.4)	2530.3 (35.1)	2530.8 (35.2)
		R	2446.7 (54.5)	2453.8 (44.3)	2443.2 (36.7)	2492.1 (36.8)
						
FUP2 (scan 3)	Amygdala	L	1371.1 (25.7)	1363.7 (27.5)	1366.1 (20.7)	1395.2 (21.7)
		R	1394.2 (24.3)	1379.2 (26.0)	1390.4 (19.5)	1403.9 (20.5)
	Hippocampus	L	2499.5 (52.6)	2457.5 (56.3)	2557.4 (42.3)	2567.8 (44.3)
		R	2448.7 (53.2)	2453.7 (56.9)	2483.4 (42.7)	2499.3 (44.8)

Abbreviations: ePNS, early persistent negative symptom; FEP, first-episode of psychosis; FUP, follow-up; L, left; R, right; sPNS, persistent negative symptoms due to secondary factors.

Mean hippocampal and amygdalar volumes are adjusted for total brain volume, age, sex and handedness, with s.e. in brackets. There were no differences in volumes between groups when looking at the data cross-sectionally.
