# Supplementary material for: Linking persistent negative symptoms to amygdala–hippocampus structure in first-episode psychosis
Source: Transl Psychiatry. 2017 Aug 8;7(8):e1195–. doi: 10.1038/tp.2017.168 (PMC5611735; doi:10.1038/tp.2017.168)
Supplement: Supplementary Material [file tp2017168x1.docx]

**Supplementary Table of Contents**

**Supplementary Methods** – Patient exclusion from neuroimaging study and quality control procedures, and MAGeT-Brain image processing

**Supplementary Table 1**- Comparison of SANS and SAPS scores across clinical timepoints using generalized estimating equations.

**Supplementary Table 2**- Linear mixed effects analysis of hippocampal and amygdalar volumes – controlling for diagnosis, antipsychotic dosages, IQ, and removing sex and handedness as covariates.

**Supplementary Figure 1**- Sample distribution by group and age.

**Supplementary Figure 2**- Example segmentation of the hippocampus and amygdala from a representative candidate.

**Supplementary Figure 3** – Three-dimensional representation of vertex-wise surface area analysis of hippocampus and amygdala.

**Supplementary Figure 4**- Negative and Positive Symptoms in FEP subgroups across clinical timepoints.

**Supplementary Figure 5** – Linear mixed effects analysis of significant amygdalar and hippocampal surface morphometry results, covarying for diagnosis, antipsychotic dosage, IQ, and removing sex and handedness as covariates.

**Supplementary Methods**

**Patient exclusion from neuroimaging study.** Forty-two patients and forty-six controls were no longer part of the neuroimaging study after baseline, due to attrition, incidental findings, substance-induced psychosis, and for other diagnostic reasons that no longer met criteria for inclusion in PEPP. This left a total of 100 patients and 48 healthy controls with at least two scans. Three patients were not included in subsequent analysis due to insufficient longitudinal symptom data (2 cases) or non-compliance to the time line of the study (1 case). Please see the section on **Quality control** **procedures** below for information on subjects who passed post-processing quality control.

**Quality control procedures.** All raw T1-weighted scans were visually inspected and quality controlled by three independent raters and scans were excluded if they exhibited excessive movement within the scanner, or the scan contained incidental findings. All raw scans that passed initial QC were then submitted to the MAGeT pipeline. All resultant segmentations and surfaces were visually inspected before analysis using Display (https://github.com/BIC-MNI/Display). Six participants (4 controls [12 scans total], 2 FEP patients [5 scans total]) were excluded entirely, and one follow-up scan belonging to a FEP patient was dropped from analysis, due to significant errors in resultant segmentations (largely due to motion and poor tissue contrast), or due to incidental findings.Note, hippocampal subfields are available in the atlas used, but given the difficulty in resolving these substructures with 1.5T MRI, only whole hippocampal volumes were considered in analyses.

**MAGeT-Brain image processing.** Raw T1-weighted scans were submitted to the MAGeT-Brain pipeline, based on a multi-atlas segmentation approach. This technique utilizes a limited number of high-resolution atlases that have been manually segmented as described previously for the amygdala (1) and hippocampus (2) (<https://github.com/CobraLab/atlases>). Segmentations are extracted from pre-defined high resolution atlases onto a library of subjects, as described in the main manuscript. Labels from the atlases were then propagated to a subset of 21 subjects (templates) from the neuroimaging study, a number shown to be optimal in previous work (3). The templates comprised a representative mix of eleven FEP patients and ten controls, and an approximately equal male to female ratio. Customization of atlas labels to templates was conducted using a nonlinear transformation with a version of Automatic Normalization Tools (ANTS) compatible with the minc-toolkit (https://github.com/vfonov/mincANTS). Next, a bootstrapping procedure was applied to final segmentations. Candidate labels were fused for each subject using a majority vote procedure (i.e. the label occurring most frequently at a specific location is retained) (4, 5).

To determine shape, surface-based representations of the amygdala and hippocampus were defined separately on the basis of the input atlas, using the marching cubes algorithm (6), and were morphologically smoothed using the AMIRA software package (Visage Imaging; San Diego, CA). The resulting surfaces have approximately 1200 vertices per amygdala and 1400 vertices per hippocampus. Nonlinear transformations required to map each participant to the input template were concatenated and averaged across the 21-subject template library to increase the signal to noise ratio (7). Using a similar logic as was used to extract volumes, surface-based representations were warped to fit each template, and subsequently to match each participant. This yields 21 possible surface representations per participant. To ensure homology between vertices to prior MAGeT-Brain segmentations, surface vertices were redefined using a Vornoi diagram (8). The median coordinate at each location/vertex of the amygdala or hippocampus is estimated to yield a single cohesive surface representation of each structure. Finally, surface area is represented as the sum of the surface area for each polygon in the surface, and all surface area values were blurred with a surface-based diffusion smoothing kernel of 5mm for both structures. See **Supplementary Figure 2** for example segmentations of the hippocampus and amygdala for a representative candidate, and **Supplementary Figure 3** for an overlaid mesh on a model of the left amygdala and hippocampus (<https://github.com/CobraLab/atlases)> depicting the vertices used in surface area analysis.

| **Omnibus** | | | | | | | |
| --- | --- | --- | --- | --- | --- | --- | --- |
|  |  | **Group** | | **Time** | | **Group*Time** | |
|  | N | Statistic(df) | p | Statistic(df) | p | Statistic(df) | p |
| SANS | 616 observations | χ2(2)= 91.68 | <0.0001 | χ2(6)= 37.62 | <0.0001 | χ2(12)= 38.19 | <0.0001 |
| SAPS |  | χ2(2)=48.70 | <0.0001 | χ2(6)= 7.18 | 0.305 | χ2(12)= 30.47 | 0.002 |
|  |  |  |  |  |  |  |  |
| **Descriptives and Post-Hoc** | | | | | | | |
|  | **1) ePNS** | | **2) sPNS** | | **3) non-PNS** | | post-hoc* |
|  | N | Mean (+SD) | N | Mean (+SD) | N | Mean (+SD) |  |
| **2** |  |  |  |  |  |  |  |
| SANS | 20 | 9.55 (3.33) | 29 | 8.45 (3.22) | 42 | 6.38 (3.21) | 1, 2 > 3 |
| SAPS |  | 3.70 (3.98) |  | 4.52 (3.10) |  | 2.69 (2.88) | 2 > 3 |
| **3** |  |  |  |  |  |  |  |
| SANS | 21 | 10.24 (3.02) | 29 | 8.10 (3.37) | 42 | 6.07 (3.55) | 1 > 2 > 3 |
| SAPS |  | 3.24 (3.19) |  | 4.90 (3.36) |  | 1.86 (2.32) | 2 > 3 |
| **6** |  |  |  |  |  |  |  |
| SANS | 18 | 10.22 (2.88) | 29 | 8.86 (3.01) | 39 | 4.77 (2.98) | 1, 2 > 3 |
| SAPS |  | 2.89 (2.99) |  | 6.45 (3.69) |  | 1.64 (1.89) | 2 > 1, 3 |
| **9** |  |  |  |  |  |  |  |
| SANS | 18 | 8.72 (3.06) | 27 | 8.11 (2.95) | 43 | 4.60 (3.11) | 1, 2 > 3 |
| SAPS |  | 2.83 (2.90) |  | 5.59 (4.42) |  | 1.88 (2.70) | 2 > 1, 3 |
| **12** |  |  |  |  |  |  |  |
| SANS | 20 | 9.35 (3.10) | 26 | 9.42 (3.84) | 44 | 3.61 (2.98) | 1, 2 > 3 |
| SAPS |  | 2.50 (2.21) |  | 7.35 (4.36) |  | 1.86 (2.89) | 2 > 1, 3 |
| **18** |  |  |  |  |  |  |  |
| SANS | 20 | 7.65 (3.48) | 28 | 8.04 (3.47) | 42 | 3.12 (2.89) | 1, 2 > 3 |
| SAPS |  | 2.50 (2.35) |  | 5.36 (4.10) |  | 1.50 (2.77) | 2 > 1, 3 |
| **24** |  |  |  |  |  |  |  |
| SANS | 20 | 6.55 (3.93) | 22 | 6.32 (3.09) | 37 | 3.76 (3.20) | 1, 2 > 3 |
| SAPS |  | 2.45 (2.52) |  | 3.68 (3.39) |  | 2.65 (4.32) | not significant |

**Supplementary Table 1**. Generalized Estimating Equations (GEE) Analyses and Statistics. Omnibus results for SAPS and SANS global scores are presented in the top panel, with Bonferroni-corrected p=0.025. Descriptives and post-hoc analyses are presented per clinical timepoint (outlined in left-hand column, 2/3/6/9/12/18/24-months after entry to clinic). Post-hoc analyses were considered significant at p<0.05.

Abbreviations: SANS/SAPS, Scales for the Assessment of Negative/Positive Symptoms. 1, early persistent negative symptoms (ePNS). 2, PNS due to secondary factors (sPNS). 3, Non-PNS.

| Structure | Side | Main Effect of Group | | | Group*Age Interaction | | |
| --- | --- | --- | --- | --- | --- | --- | --- |
|  |  | Statistic(df) | *q*-value | post-hoc | Statistic(df) | *q*-value | post-hoc |
|  |  | A. Covaried by Diagnosis | | | | | |
| Amygdala | L | F(2,237)=5.13 | **0.013** | 1,3<2 | F(2,237)=5.02 | **0.015** | 1,3<2 |
|  | R | F(2,237)=2.51 | 0.11 | - | F(2,237)=2.28 | 0.13 | - |
| Hippocampus | L | F(2,237)=0.27 | 0.77 | - | F(2,237)=0.12 | 0.89 | - |
|  | R | F(2,237)=5.12 | **0.013** | 1<2 | F(2,237)=5.69 | **0.015** | 1<2 |
|  |  | B. Covaried by Antipsychotic Dosage | | | | | |
| Amygdala | L | F(2,239)=5.16 | **0.013** | 1,3 <2 | F(2,239)=5.16 | **0.013** | 1,3<2 |
|  | R | F(2,239)=2.61 | 0.1 | - | F(2,239)=2.39 | 0.13 | - |
| Hippocampus | L | F(2,239)=0.49 | 0.61 | - | F(2,239)=0.32 | 0.73 | - |
|  | R | F(2,239)=5.15 | **0.013** | 1<2 | F(2,239)=5.84 | **0.013** | 1<2 |
|  |  | C. Removing Sex and Handedness as Covariates | | | | | |
| Amygdala | L | F(3,352)=3.49 | **0.04** | 1<2,4; 2<3 | F(3,352)=3.54 | **0.03** | 1<2,4; 2<3 |
|  | R | F(3,352)=1.58 | 0.25 | - | F(3,352)=1.47 | 0.29 | - |
| Hippocampus | L | F(3,352)=0.29 | 0.83 | - | F(3,352)=0.18 | 0.91 |  |
|  | R | F(3,352)=3.31 | **0.04** | 1<2 | F(3,352)=3.64 | **0.03** | 1<2,4 |
|  |  | D. Covaried by IQ* | | | | | |
| Amygdala | L | F(3,344)=3.21 | **0.046** | 1<2,4; 2<3 | F(3,344)=3.26 | **0.044** | 1<2,4; 2<3 |
|  | R | F(3,344)=1.59 | 0.25 | - | F(3,344)=1.46 | 0.31 | - |
| Hippocampus | L | F(3,344)=0.16 | 0.92 | - | F(3,344)=0.10 | 0.96 |  |
|  | R | F(3,344)=3.23 | **0.046** | 1<2 | F(3,344)=3.64 | **0.044** | 1<2,4 |

**Supplementary Table 2.** Linear mixed effects analyses comparing FEP patient subgroups, including different covariates in addition to covariates presented in main manuscript: i.e. sex, handedness, and total brain volume (and age for main effect of group). Antipsychotic dosage was calculated as cumulative antipsychotic medication prescribed (converted to chlorpromazine equivalent dosage in mg), and multiplied by medication adherence. Medication adherence [0=never (0%), 1=very infrequently (1% to 25%), 2=sometimes (26% to 50%), 3=quite often (51% to 75%), 4=fully (76% to 100%)] was determined using a validated protocol based on com­posite information obtained from the patient, family members, and treating team and has been shown to be as efficacious as pill-counting (9). Note, controls were not included in analyses A and B, as controls do not have diagnostic/antipsychotic medication information. Analyses C and D include controls. An FDR correction for multiple comparisons was applied, with significant q-values<0.05 bolded. All post-hoc analyses correspond to p<0.05.

Abbreviations: L, Left. R, Right. 1, early persistent negative symptoms (ePNS). 2, PNS due to secondary factors (sPNS). 3, non-PNS. 4, Controls.

* Note for analyses D (controlling for IQ), IQ information is missing for two controls, and thus they were excluded from this particular analysis.


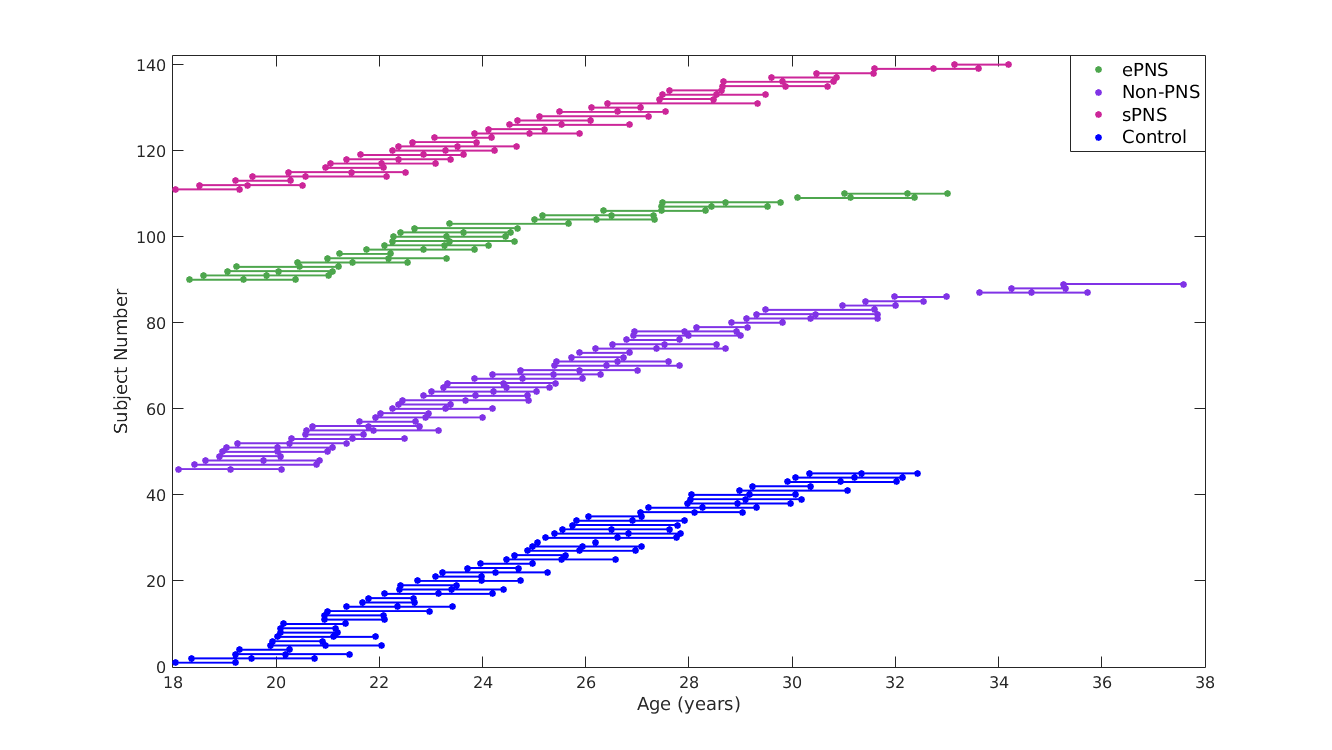


**Supplementary Figure 1**. Sample distribution by group and by age. Each horizontal line represents a subject, with each point corresponding to a single scan.

**X=58 Y=135 Z=132**

**
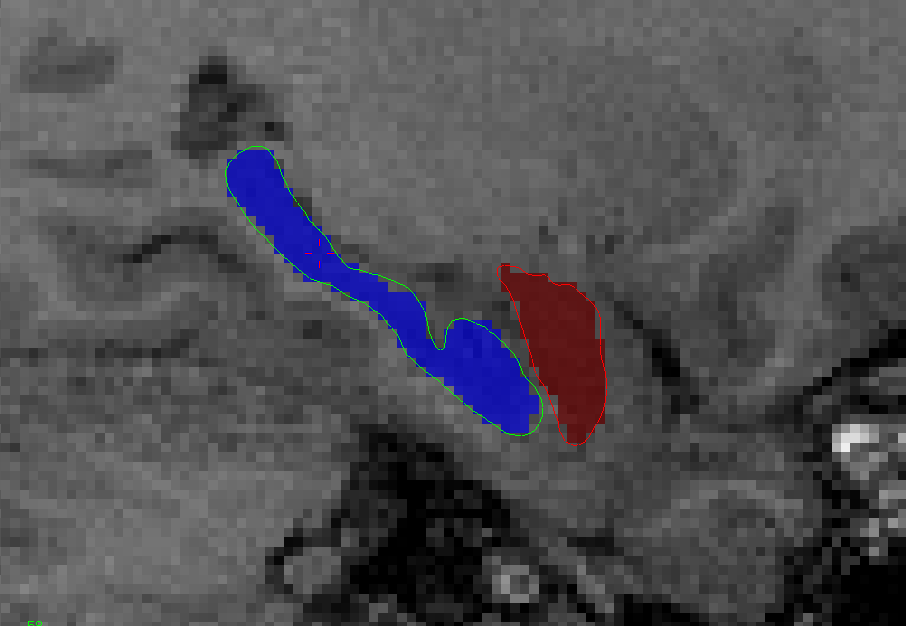

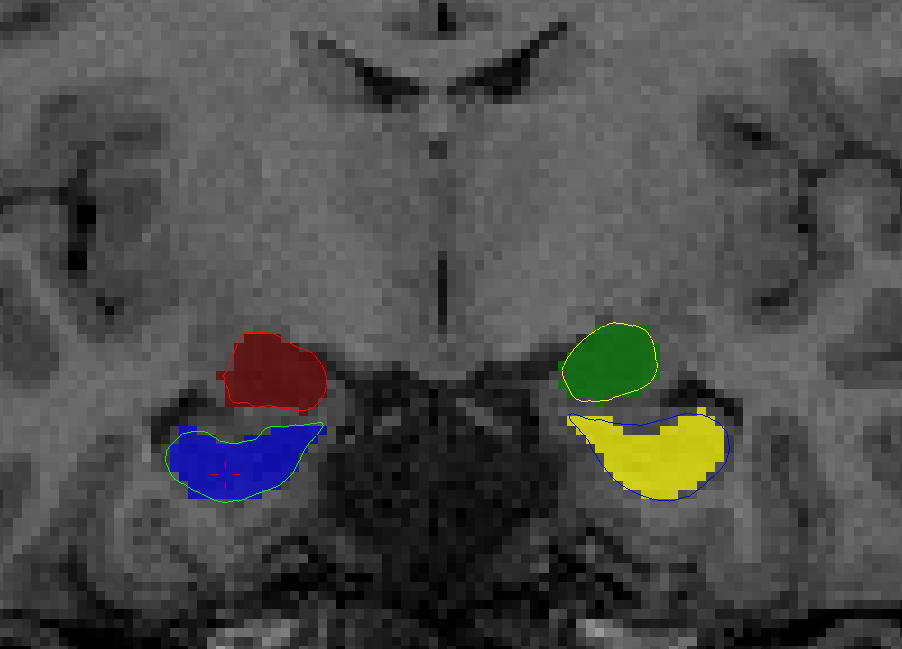

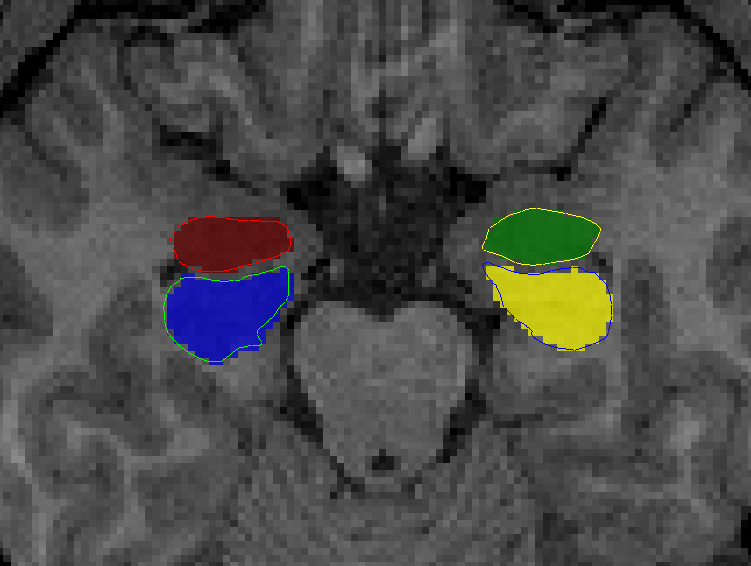
**

**Supplementary Figure 2.** Example segmentation of bilateral hippocampus and amygdala and corresponding surfaces from the MAGeT-Brain algorithm on a representative subject. Legend: Blue, Left hippocampus. Yellow, Right Hippocampus. Red, Left Amygdala. Green, Right Amygdala. Corresponding surfaces are outlined around each label. X, Y, and Z coordinates listed above each image corresponding to section in sagittal, coronal, and horizontal views in MNI standard space, respectively.


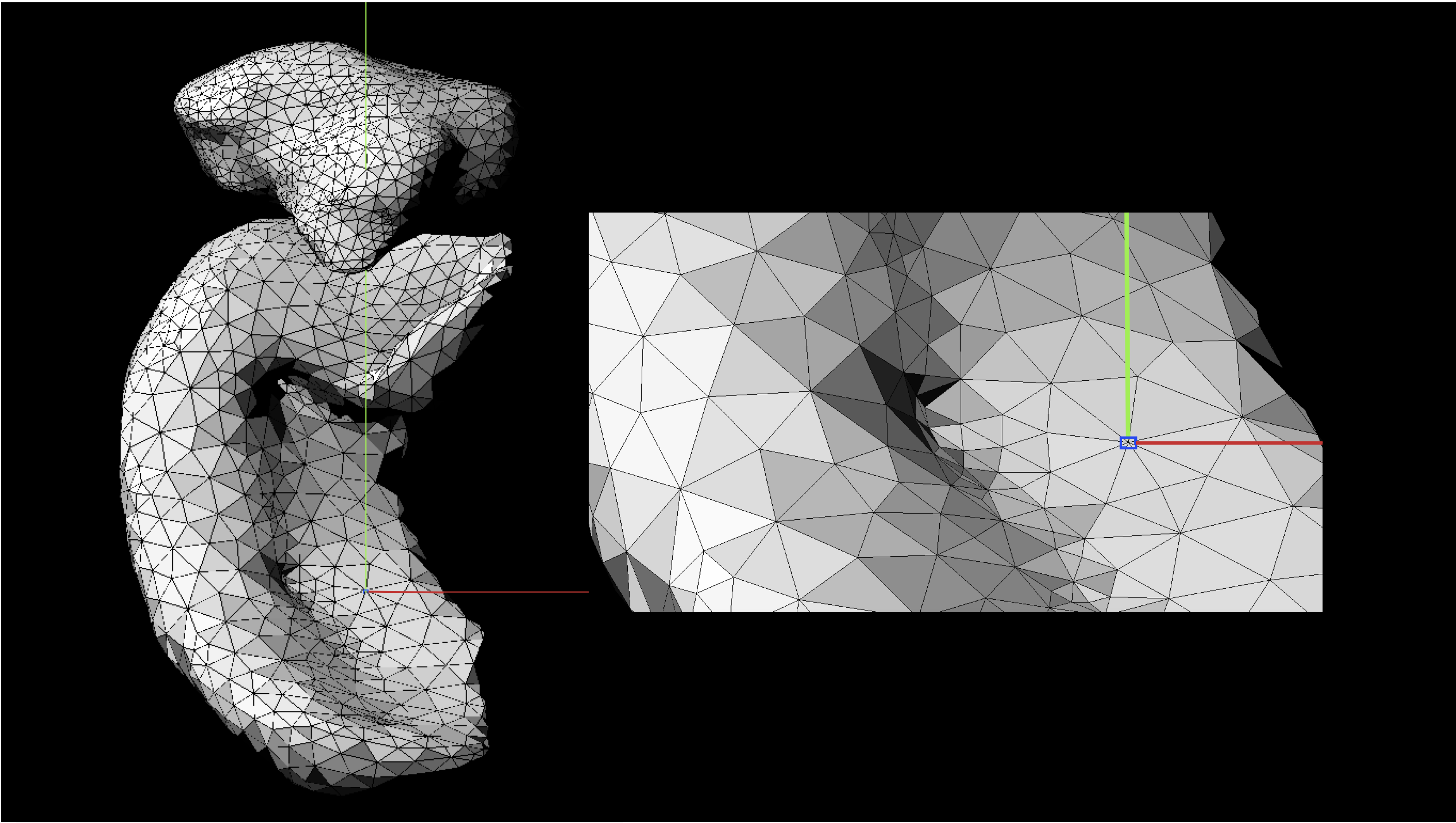


**Supplementary Figure 3.** Three-dimensional mesh overlaid on a model surface of the amygdala and hippocampus. The dorsal view of the left amygdala and hippocampus is depicted on the left. The right is a magnified version to better visualize the polygons comprising one particular vertex, with the crosshairs pointing to the same vertex in both images.


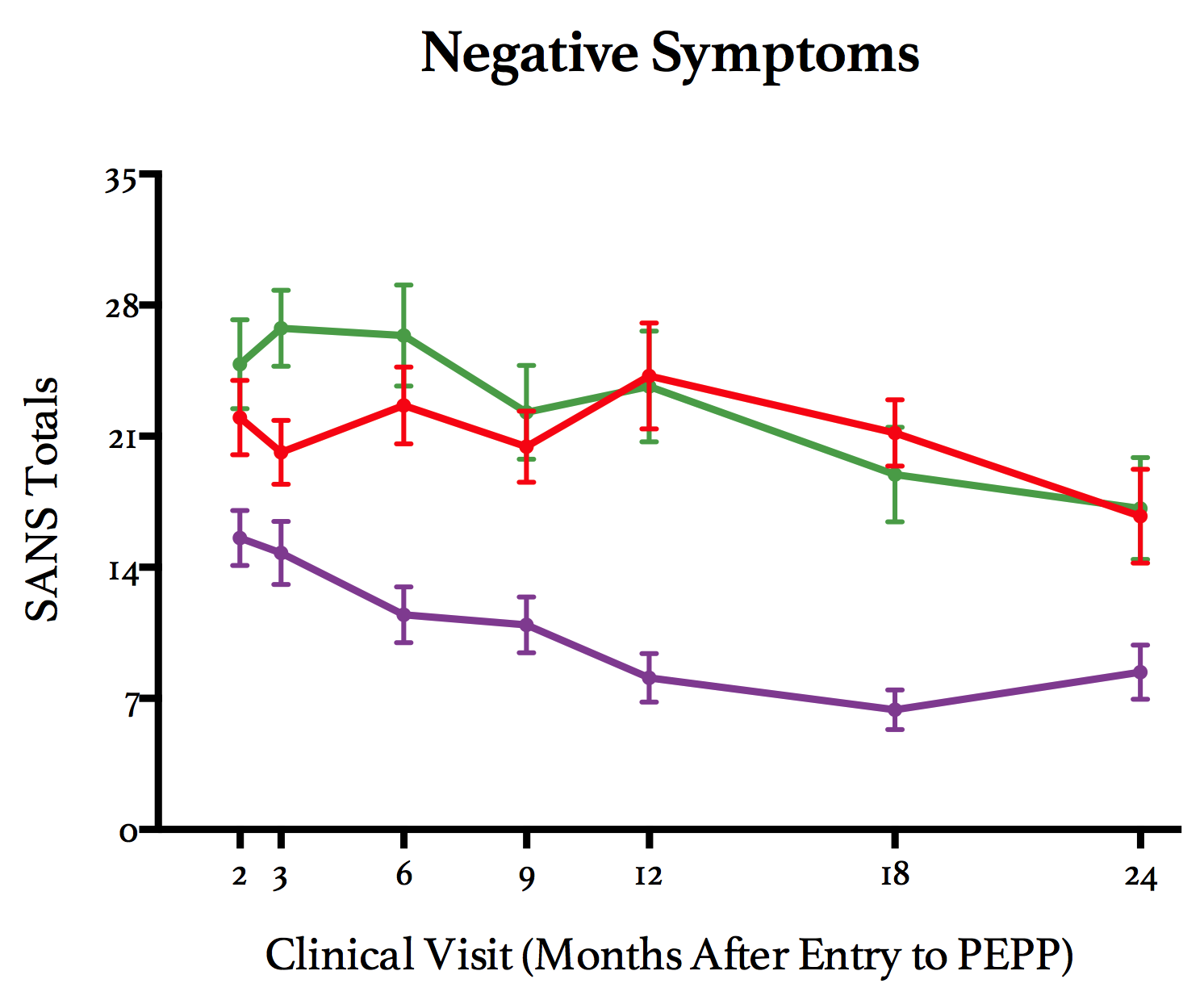

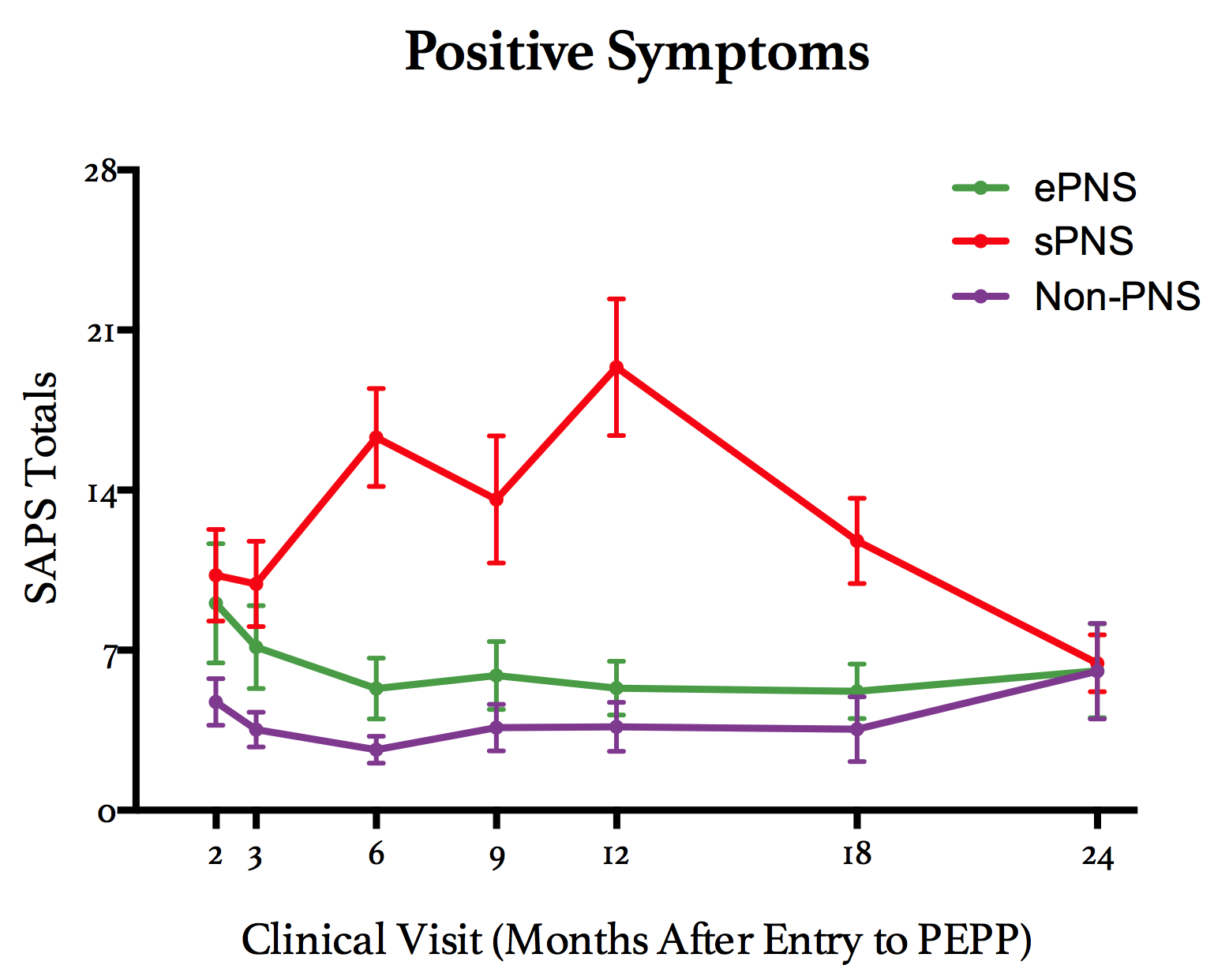


**Supplementary Figure 4**. Negative and Positive symptoms, as assessed by the SANS and SAPS, respectively, across each clinical timepoint for the three FEP subgroups. Corresponding statistics are in **Supplementary Table 1**.

Abbreviations: SANS/SAPS, Scales for the Assessment of Negative/Positive Symptoms. ePNS, early persistent negative symptoms. sPNS, persistent negative symptoms due to secondary factors.


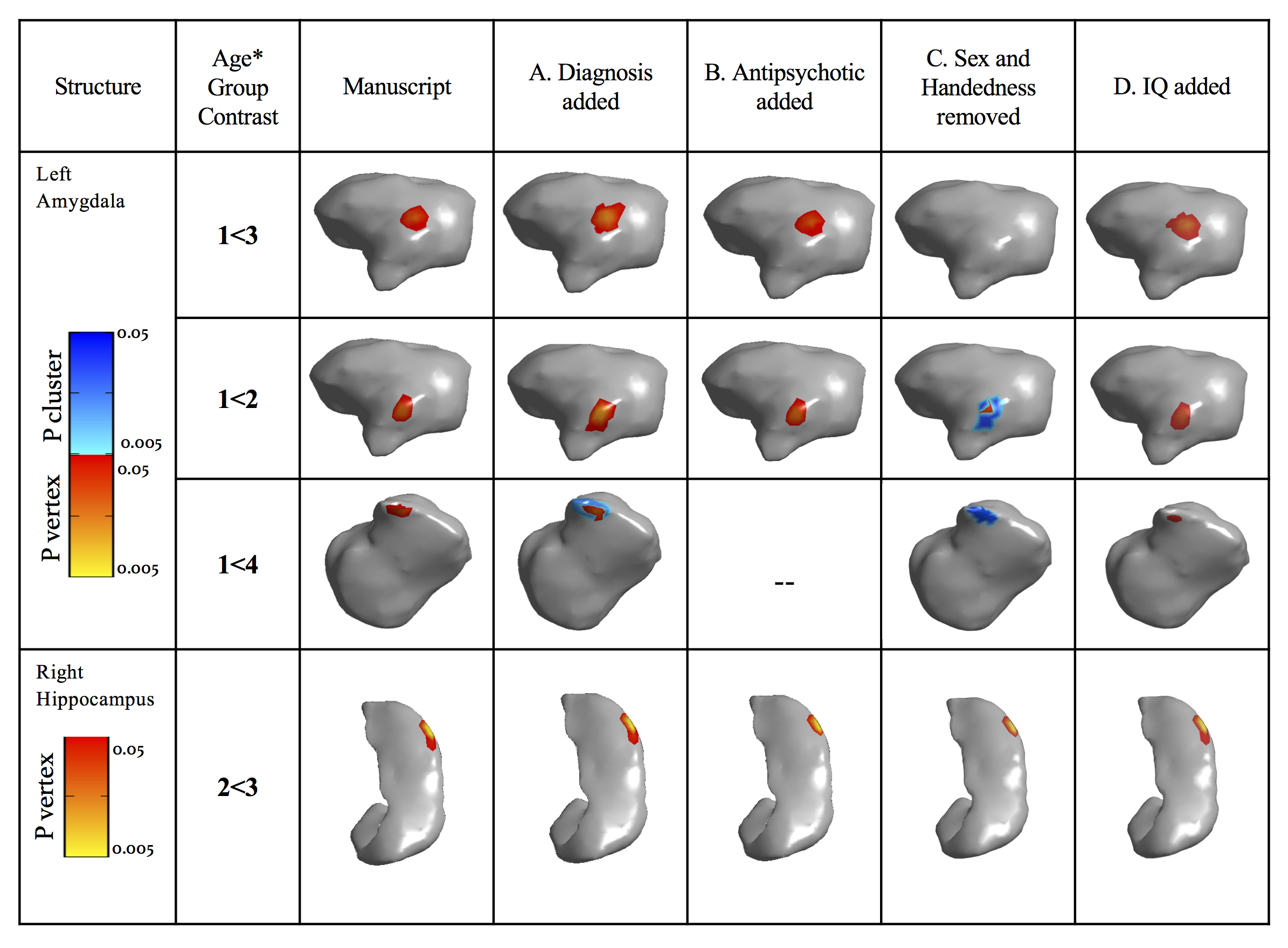


**Supplementary Figure 5.** Linear mixed effects analyses of surface area differences for age*group interaction, including different covariates in addition to covariates presented in main manuscript: i.e. sex, handedness, and total surface area of each structure examined (with the exception of **analyses C** where sex and handedness are removed). Antipsychotic dosage was calculated as cumulative chlorpromazine equivalent dosage in mg, multiple by percent medication adherence. Contrast for controls vs. ePNS (1<4) covarying for antipsychotic dosage was not included given that controls do not take medication. First two rows depict dorsal view of left amygdala, third row depicts posterior view of left amygdala, and bottom fourth row depicts ventral view of right hippocampus. Statistical maps are thresholded with random field theory (RFT). Note, one cluster did not survive correction for multiple comparisons with RFT after removing sex and handedness as covariates, namely when comparing age trajectories between ePNS and NonPNS patients in the left central amygdala, although this cluster nearly reached statistical significance.

Abbreviations: 1, early persistent negative symptoms (ePNS). 2, PNS due to secondary factors (sPNS). 3, Non-PNS. 4, Controls.

**SUPPLEMENTARY REFERENCES**

1. Treadway MT, Waskom ML, Dillon DG, Holmes AJ, Park MT, Chakravarty MM, et al. Illness progression, recent stress, and morphometry of hippocampal subfields and medial prefrontal cortex in major depression. Biol Psychiatry. 2015;77(3):285-94.

2. Winterburn JL, Pruessner JC, Chavez S, Schira MM, Lobaugh NJ, Voineskos AN, et al. A novel in vivo atlas of human hippocampal subfields using high-resolution 3 T magnetic resonance imaging. Neuroimage. 2013;74:254-65.

3. Pipitone J, Park MT, Winterburn J, Lett TA, Lerch JP, Pruessner JC, et al. Multi-atlas segmentation of the whole hippocampus and subfields using multiple automatically generated templates. Neuroimage. 2014;101:494-512.

4. Collins DL, Holmes, C. J., Peters, T. M. and Evans, A. C. Automatic 3-D model-based neuroanatomical segmentation. Hum Brain Mapp. 1995;3:190-208.

5. Collins DL, Pruessner JC. Towards accurate, automatic segmentation of the hippocampus and amygdala from MRI by augmenting ANIMAL with a template library and label fusion. Neuroimage. 2010;52(4):1355-66.

6. Lorensen WE CH. Marching cubes: a high resolution 3D surface construction algorithm. . Proceedings of the 14th Annual Conference on Computer Graphics and Interactive Techniques. 1987;New York, NY, USA.

7. Borghammer P, Ostergaard K, Cumming P, Gjedde A, Rodell A, Hall N, et al. A deformation-based morphometry study of patients with early-stage Parkinson's disease. Eur J Neurol. 2010;17(2):314-20.

8. Lyttelton OC, Karama S, Ad-Dab'bagh Y, Zatorre RJ, Carbonell F, Worsley K, et al. Positional and surface area asymmetry of the human cerebral cortex. Neuroimage. 2009;46(4):895-903.

9. Cassidy CM, Rabinovitch M, Schmitz N, Joober R, Malla A. A comparison study of multiple measures of adherence to antipsychotic medication in first-episode psychosis. J Clin Psychopharmacol. 2010;30(1):64-7.
